# Differential neural response to psychoanalytic intervention techniques during structural interviewing: A single-case analysis using EEG

**DOI:** 10.3389/fnhum.2022.1054518

**Published:** 2023-01-04

**Authors:** Anna Buchheim, Otto F. Kernberg, Nikolaus Netzer, Peter Buchheim, Corinna Perchtold-Stefan, Barbara Sperner-Unterweger, Fabian Beckenbauer, Karin Labek

**Affiliations:** ^1^Institute of Psychology, University of Innsbruck, Innsbruck, Austria; ^2^New York-Presbyterian Hospital–Westchester Division, New York, NY, United States; ^3^Hermann Buhl Institute, University of Innsbruck, Eurac Research, Innsbruck, Austria; ^4^Eurac Research, Bolzano, Italy; ^5^Department of Psychosomatic Medicine and Psychotherapy, Technical University of Munich, Munich, Germany; ^6^Institute of Psychology, University of Graz, Graz, Austria; ^7^Department of Psychiatry, Psychotherapy and Psychosomatics, Medical University Innsbruck, Innsbruck, Austria

**Keywords:** structural interviewing, psychoanalytic intervention techniques, clarification, confrontation, interpretation, EEG analysis, alpha power

## Abstract

**Background:**

Previous studies detected changes in the electroencephalographic (EEG) signal as an effect of psychoanalytic interventions. However, no study has investigated neural correlates of specific psychoanalytic interventions in the EEG power spectrum yet. In the present case study, we contrasted three types of interventions (clarification, confrontation, and interpretation) and a neutral control condition during a structural psychoanalytic interview conducted while EEG was recorded.

**Methods:**

A 27-year-old male patient diagnosed with major depressive disorder and borderline personality disorder with recurrent suicidal and self-injurious behavior underwent a structural interview while recording EEG. Two independent experts selected by consensus the characteristic episodes of the four conditions (clarification, confrontation, interpretation, and neutral control) within the interview, which were included in the EEG analyses. Fast Fourier transformation (FFT) was applied to subsegments of the intervention type to analyze the EEG power spectra. Alpha and beta power from central, frontal, and parietal sites were considered in linear mixed-effects models with segments as a random factor with maximum-likelihood estimates due to the lack of balance in the length of the interview segments.

**Results:**

The interventions “interpretation” and “confrontation” showed a significantly lower alpha power compared with the control condition in the central electrodes. In the frontal and parietal sites of the alpha power and all beta power sites, the omnibus tests (full model/model without intervention) and comparisons relative to control conditions showed no significant overall result or failed significance after alpha error correction.

**Conclusion:**

Incisive interventions, such as confrontation with discrepancies and interpretation of unconscious intrapsychic conflicts, may have provoked temporary emotional lability, leading to a change in psychic processing akin to interference from external stimuli. This conclusion is consistent with the finding that interpretations, which are potentially the most concise interventions, had the strongest effects on alpha power. Using EEG during therapeutic psychoanalytic intervention techniques might be a helpful tool to evaluate differential responses to the psychotherapeutic process on a neural level. However, this single-case result has to be replicated in a larger sample and does not allow generalizations.

## 1. Introduction

### 1.1. Basic principles of psychoanalytic treatment

Psychoanalytic psychotherapies pursue change strategies related to unconscious conflicts, defensive processes, personality functioning, and structure, as well as typical maladaptive behavior patterns. They refer to the identification and processing of dysfunctional relational, experiential, and behavioral patterns of the therapeutic relationship and the processes of transference and countertransference between a therapist and a patient (Thomä and Kächele, 1985). From a psychoanalytic perspective, it is assumed that it is not primarily the elaboration of an alternative behavioral strategy that leads to personality change but rather the ability to reflect on and re-evaluate the underlying problems of behavior and of contradictory self-representations that creates the conditions for lasting change. The psychodynamic focus is either on more restricted abilities for self-regulation and relationship regulation or on the inability to recognize, understand, reflect, and mentalize one’s inner processes and those of others (Kernberg, 1999; Bateman and Fonagy, 2004).

Hereby, four aspects jointly constitute the very essence of psychoanalytic techniques: Interpretation, transference analysis, technical neutrality, and countertransference analysis (Etchegoyen, 1999; Kernberg, 2016). Interpretation is defined as a verbal communication by an analyst describing the hypothesis of an unconscious conflict that seems to have dominantly emerged in the patient’s communication in the therapeutic encounter. The interpretative process may be classified into clarification, confrontation, and interpretation. Clarifications and general questions rely on the material more explicitly mentioned in the patient’s narratives (conscious or preconscious), which is associated with conscious processes. By contrast, the defining characteristic of confrontation and interpretation refers to the unconscious or preconscious material. Confrontations pave the way for analytic work, as they draw attention to important topics, indicate potential similarities (or inconsistencies) in patient narratives, and test the ability of the patient to initiate a process of self-reflection. Interpretations offer possible hypotheses beyond what the patient already knows and may unravel a patient’s conflict or uncover motives of behavior. This condensed hypothesis is that interpretation “in the here and now,” to be followed or completed with interpretation “in the there and then,” that is, the genetic aspects of interpretation that refer to the patient’s past linking the unconscious aspects of the present with the unconscious aspects of the past. Transference may be defined as the unconscious repetition in the here and now of pathogenic conflicts from the past, and the analysis of transference is the main source of specific change brought about by psychoanalytic treatment (Kernberg, 2016).

There is a range of manualized psychoanalytic therapies that vary in the extent to which they focus on more supportive or interpretive elements (e.g., transference-focused psychotherapy by Kernberg et al. (1989), Clarkin et al. (1999), panic-focused psychodynamic psychotherapy by Milrod et al. (1997), Mentalization Based Treatment by Bateman and Fonagy (2016). These manualized approaches share the core meaning of a comprehensive initial diagnostic assessment for treatment planning. Patients with more severe disturbances in personality functioning require more highly structured treatment settings, and frequently, the establishment of a formal treatment contract is necessary to minimize destructive attacks against self, others, and the treatment. Clinical interviews focusing on personality functioning, including several domains such as self and interpersonal functioning as the central tenet of personality pathology, provide a fruitful diagnostic approach. This is shared by many theoretical orientations and evidence-based treatments (Pincus et al., 2020).

### 1.2. The structural interview as a diagnostic tool to assess personality structure and functioning

In structural interviewing, Kernberg (1981, p 169) explained: “structural diagnosis as an overall intrapsychic organization that provides stability, continuity throughout time and is facilitated by a special type of clinical interview—a ‘structural interview’—that focuses sharply on the relation between the interaction of patient and interviewer, the patient’s interpersonal functioning in general and the history of the present illness.” In severe personality disorders, Kernberg (1984) focused on the structural characteristics of Borderline Personality Organization. In psychodynamic therapy for personality pathology (Caligor et al., 2018), the structural interview is divided into the following: Phase I: Presenting complaints and symptoms, phase II: Personality functioning, phase III: Identity formation, phase IV: Past history, and final phase: Outstanding issues and questions. The structural interview stands for a pioneering, integrative approach in which, besides psychopathology and psychodynamics, personality structure and functioning on a continuum of severity of pathology on different levels of personality organization can be assessed (Buchheim et al., 1987). It combines psychopathology with a psychoanalytic focus on the patient–interviewer interaction. In the initial phase, the interviewer presents four questions in sequence about the main complaints and problems, their development, expectations of treatment, and current state of health and focuses on the clarification of the symptoms, the pathological character traits, and the conflicts or difficulties offered by the patient. In the middle phase, the interviewer asks the patient to characterize himself and describe the important reference persons, to get a picture of the internalized self and object relations, to understand the extent of identity diffusion or the capacity for identity integration, and to understand the typical defense mechanisms. The aim of the interview was also to gain a comprehensive picture of the patient’s essential areas of life, such as work, leisure, family, partnership, and sexuality. In addition, the ability to do a reality check to have a distinction between psychotic symptoms and disorders is always assessed.

By focusing on the interaction between the patient and the interviewer with the help of the psychoanalytic techniques of *clarification*, *confrontation*, and *interpretation* of conflictual themes and defense mechanisms, it is possible to work out the symptomatology and the underlying personality structure at the same time. A salient feature of the structural interview is a circular approach to questioning. Repeatedly returning to previously expressed but not yet sufficiently clarified symptoms and problems allows the interviewer to return to the starting point and reinitiate a new circle of inquiry, evaluating contradictions in different contexts at a later time and forming a more complete picture.

The structural diagnosis depends upon how the patient reacts and handles clarifications, confrontations, and interpretations in the interview. This interview technique is a challenge for both interviewer and patient and can lead to a kind of “experimental labilization,” through which the lowered functional level of the personality organization (“bottom of the rock”) under stressful conditions can also become recognizable in the interview.

The focus on the severity of self- and interpersonal dysfunction represents a considerable step forward in making diagnoses clinically meaningful (Blüml and Doering, 2021). With the alternative model, the DSM-5 recognizes the need for a dimensional approach to personality pathology and functioning, which posits self and interpersonal functioning as key defining dimensions of both normal personality and PDs, along a continuum of severity of dysfunction in these domains (Hörz-Sagstetter et al., 2018). This new development underlines the lasting topicality of Kernberg’s theoretical approach.

### 1.3. Research on psychoanalytic constructs and effects of treatment

There is strong, accumulating evidence from attachment theory, experimental psychology research, including neuroscience, and psychotherapy research that support the validity and clinical usefulness of several psychoanalytic basic constructs (e.g., defensive processes, transference and countertransference, insight, and mentalizing) that underlie psychodynamic psychotherapy (Yakeley, 2018; Levy et al., 2019).

Recently, three trials of psychodynamic therapies were structured to focus predominantly on the therapeutic relationship and transference. The first experimental study of transference compared psychodynamic therapy by randomizing patients to receive or not receive transference interpretations (Høglend et al., 2008). In the subsample of patients with poor personality functioning (predominantly cluster C personality disorders), therapy without transference interpretations was less effective at improving patients’ insights into their conflicts and patterns of defense, leading to worse improvements in psychosocial functioning compared to therapy with consistent use of transference interpretations (Høglend et al., 2008, 2011). Consistent with the integrative view for individuals with lower levels of personality functioning, transference interpretations were especially helpful if the therapists acted from a “parental” stance, whereas the contrary was true for individuals with higher levels of personality functioning. Among samples of patients with borderline personality disorder (BPD), transference-focused psychotherapy (TFP) has been compared to dialectical-behavioral therapy, dynamic-supportive therapy, and treatment by community experts (Clarkin et al., 2007; Doering et al., 2010). In both trials, TFP was also shown to uniquely promote patient improvements in mentalization and attachment security (Levy et al., 2006; Fischer-Kern et al., 2015; Buchheim et al., 2017), which is consistent with a view that transference interpretation may be uniquely helpful in this population specifically for fostering intrapsychic integration.

Recent developments in neuroscience have fertilized and intensified an interdisciplinary dialogue between psychoanalysis and neuroscience (e.g., Kandel, 1998, 2013; Carhart-Harris and Friston, 2010; Prosser et al., 2018; Solms, 2021a,b). The cooperation between the two disciplines has resulted in numerous experimental studies that shed new light on psychoanalytic constructs and techniques (e.g., Solms, 2011; Panksepp and Solms, 2012; Böker et al., 2013; Shevrin et al., 2013). One study group recently focused on neural responses on free association in healthy individuals (Kehyayan et al., 2013; Schmeing et al., 2013). Moreover, several fMRI studies demonstrated the effects of psychodynamic treatment on a neural level (Beutel et al., 2010, 2012; Buchheim et al., 2012; de Greck et al., 2011, 2013; see review Abbass et al., 2014; Messina et al., 2016; Perez et al., 2016). Only a few studies examined neural changes during psychodynamic treatment using electroencephalographic (EEG) (Unterrainer et al., 2013, 2014; Buchheim et al., 2018). Buchheim et al. (2018) demonstrated that, at the beginning of the treatment, patients showed significantly higher late positive potentials (LPPs) at the frontocentral sites and sustained gamma-band activity compared to the controls. After 15 months of treatment, LPP amplitudes and gamma-band responses of the patients decreased and equalized to the amplitudes of the healthy controls. Here, LPP and gamma-band activity were considered potential endophenotypes of the processing of emotional content in the course of psychoanalytic treatment.

However, no study so far has investigated the neural responses to basic psychoanalytic interventions (clarification, confrontation, and interpretation) in a standardized interview setting. For the study of awake humans, alpha, beta, and gamma waves were central frequency bands observed in various tasks. For example, alpha rhythms (8–12 Hz) are highly responsive to sensory stimuli and motor tasks (Klimesch et al., 1996; Williamson et al., 1997; Cohen, 2017). Beta oscillations (13–30 Hz) have been identified in many perceptual, cognitive, and motor processes in various EEG studies (e.g., Schmidt et al., 2019). Gamma-band waves were excluded from further analyses to carefully account for possible confounds with muscle artifacts occurring in a similar frequency range. The aim of the present study was to analyze for the first time a patient’s brain activity during a structural interview by contrasting clarification, confrontation, and interpretation techniques using EEG.

## 2. Materials and methods

The patient and the therapist gave written informed consent to the analysis and publication of the data. However, the case report was anonymized to protect the patient’s identity. The case study was approved by the ethical review board of the University Innsbruck. The structural interview was administered by Otto Kernberg, the senior expert in the interviewing procedure, who had no information about the patient.

### 2.1. Participant

The 27-year-old male patient was diagnosed with major depression with past suicidal attempts and was in inpatient treatment. The patient has lost his father, who committed suicide 6 years ago. The patient associated the beginning of his symptoms (depressive mood, self-mutilating behavior, suicidal thoughts, and feelings of emptiness) with severe loss. Since then, the patient has lost all his interests, broke off his studies at university, and had relationship problems with significant others. His temporary promiscuous behavior especially endangered his relationship with his girlfriend. Before the start of the interview procedure, the patient and the therapist agreed to participate in the study. Neither the therapist nor the patient had any information about each other and did not receive payment for conducting or participating the interview. The interview was assessed in the initial phase of the inpatient treatment. Both were given general information about the study and signed a declaration of their willingness to participate in the interview and the EEG recordings.

### 2.2. Interview procedure

The interview was conducted in the standardized format described by Kernberg (1981). After a multi-part initial questionnaire about the main complaints and problems, their development, expectations of treatment, and current state of health, the interviewer, Kernberg, initially focused on the symptoms, conflicts, or difficulties offered by the patient. The interviewer *clarified* to understand the main symptoms like depressive mood, strong self-injury, and concentration problems. He continued to *clarify* if there were any other problems besides the depressive symptoms and self-harm. As a result, the patient reported the suicide of his father 6 years ago with no feelings of mourning but of being shocked. The next clarification process focused on the concentration problems in his studies and his failure to succeed in examinations. Along with the circular approach to questioning, the interviewer again *clarified* if there were any other problems besides the reported ones. The patient first denied having any other problems several times but then reported the negative effects of his self-harm behavior on his long-lasting relationship with his girlfriend. In this context, he finally confessed that he had cheated on her with other women, which had led to an ongoing severe crisis by the time of the interview. In the further course of the interview, the interviewer asked the patient to characterize himself and his girlfriend to get a picture of the internalized self and object relations and the extent of identity diffusion. Here, the first contradictions occurred: On the one side, the girlfriend was described as available and empathic, with a relationship in which both could share a lot, including satisfying sexuality. On the other side, the patient reported about the senselessness in his life with no support from anyone, including his parents. The interviewer now *confronted* him several times that he denied the existence of his girlfriend and that he prefers to harm himself instead of studying. He confronted the patient with his way of mourning by being shocked but without any feelings of grief. The patient then realized that he felt abandoned by his father. The interviewer again confronted the patient that he was going to destroy his relationship by cheating on his girlfriend and that he was in danger of being abandoned. The patient could see this point and showed adequate feelings of guilt. In the next step, the interviewer summarized the core conflicts of the patient by confronting him again and giving several interpretations. He interpreted that the patient unconsciously did not dare to have a better life than his father and therefore is at risk of destroying himself and his relationships, especially the one with his girlfriend. He interpreted his lack of concern for himself and his inability to make any choices for change as a suboptimal compromise to avoid competing with his father, who was leading a most unhappy life. The patient was emotionally affected by these numerous interpretations and showed signs of insight and an increasing wish to change some major aspects of his life.

This interview technique led to the “experimental labilization” (Kernberg, 1981) described earlier, through which the lowered functional level of personality organization (borderline personality organization) with clear signs of identity diffusion, a disrupted concept of self, lack of care, concern, and responsibility for himself and others became evident under these activating stressful conditions in the interview.

### 2.3. EEG procedure

Before the interview, EEG electrodes were placed on the patient’s head. The patient’s EEG signal was measured continuously during the structural interview. During the interview, the patient and therapist sat facing each other. To achieve an exact match with the EEG signal, an audiovisual recording of the EEG and the interview was made simultaneously. The brain activity was recorded throughout the whole duration of the interview. Thus, the entire EEG recording lasted 63 min 35.7 s. To reduce possible artifacts, we used only the patient’s EEG signal when the patient was not talking but paying attention to the therapist’s treatment. Recording, preprocessing, and data analysis were performed using Brain Vision Recorder software (2.0, Brain Products, Gilching, Germany) and Brain Vision Analyzer software (2.0, Brain Products, Gilching, Germany).

### 2.4. Expert ratings of the therapeutic interventions

To evaluate the therapist’s interventions during the clinical interview, the entire interview was transcribed in the first step. The transcript was then used to assess the type of intervention (clarification, confrontation, interpretation, and the control condition) by two independent and experienced experts in the field. A joint consensus then finalized the results of the expert’s assessments. The final and definitive assessments of the interventions were then included in the EEG analyses. Overall, the interview resulted in 28 interview sequences for confrontation, 91 for clarification, seven for interpretation, and 27 for the control condition. This resulted in 218 EEG segments for confrontation, 434 for clarification, 71 for interpretation, and 79 for control segments. For the control condition, neutral sentences or statements without emotional content were selected, such as “What does middle school mean?” or “My name is Prof. Kernberg, I am a psychiatrist from New York.” These segments have been used as a basis for the EEG analyses.

### 2.5. Acquisition and processing of EEG data

Electroencephalographic (EEG) activity was recorded at 30 electrode sites of the extended 10–20 system with vertical and horizontal electrooculogram (EOG) components. A set of 11 silver electrodes attached with a glue paste (Nihon Kohden Elefix EEG paste) to the scalp was used to record EEG signals. The impedances of the EEG electrodes were below 5 kΩ. EEG data were sampled at 2,500 Hz and subsequently down-sampled to 2,048 Hz. EEG data were carefully checked for artifact-contaminated signals (eye blinks, horizontal and vertical eye movements, muscle artifacts, etc.) by visual inspection. For further processing of the data, an average offline reference was computed. The data were filtered by excluding fluctuations below 1 Hz (time constant 3.0 s, 48 dB/oct) and above 45 Hz (48 dB/oct).

Based on the therapist’s interventions, we divided the patient’s artifact-free epochs of the EEG signal into “segments” according to intervention type: Clarification, confrontation, interpretation, and a control condition. These segments were further divided into non-overlapping subsegments of duration 1 s for spectral analysis. For artifact-free epochs, EEG power spectra (within a frequency range of 1–45 Hz) were computed using a fast Fourier transformation (FFT) on the subsegments of 1 s with a maximum resolution of 1 Hz after applying a 10% Hanning window. The signal sequence in each channel of the EEG data was decomposed into four specific spectral bands (delta: 0.5–3.5 Hz, theta: 4–7.5 Hz, alpha: 8–12.5 Hz, and beta: 13–30 Hz). Only the alpha and beta bands were considered in the statistical analysis.

### 2.6. Statistical analysis

Logarithms of alpha and beta power μV^2^ in frontal (F: Fp1 and Fp2), central (C: C3 and C4), and parieto-temporal (PT: P3 and P4) electrodes were taken and modeled as a function of intervention type (clarification, confrontation, interpretation, and the control condition) and laterality in a linear mixed-effects model with segments as a random factor. Since the interview segments differed in length, we relied on maximum-likelihood estimates of random effects to account for the lack of balance in the data (Gelman and Hill, 2006; Twisk, 2006). We adopted a Bonferroni-corrected significance threshold for the three electrode sites in omnibus χ^2^-tests of the effect of intervention type (*p* < 0.017). When the null could be rejected in the omnibus test, we followed up with planned comparisons of the intervention types relative to the control condition (two-tailed). The analysis was conducted using the freely available package R (The R Foundation for Statistical Computing,^1^ Vienna, Austria; repeated measures regression: function lme, package nlme, Pinheiro and Bates (2000), mixed-effects models in S and S-PLUS. Berlin: Springer.). Boxplots were drawn with the freely available package ggplot2 (Wickham, 2016, Berlin, Springer).

## 3. Results

### 3.1. Clinical evaluation of the interview by the therapist

The 27-year-old male patient was diagnosed with major depression and depressive personality disorder. The structural interview came to the structural diagnosis of a “borderline personality organization.” At this level, reality testing is intact. However, patients with a borderline level of personality organization have a fragmented sense of self and others (unlike the less severe neurotic organization with an integrated self). Because they possess a fragmented sense of self, they do not have a consistent view of themselves or others over time and across situations. This fragmented sense of self is the most significant and defining feature of the borderline level and results in severe and repetitive problems with interpersonal relationships. In our case, the patient has lost his father, who committed suicide 6 years ago. The patient’s fragmented sense of self and others became evident in that he could not mourn at all but was shocked at the same time. Moreover, the patient associated the beginning of his symptoms (depressive mood, self-mutilating behavior, suicidal thoughts, and feelings of emptiness) with this loss. Since then, the patient shown a lack of engagement at the university, a loss of interest, and developed relationship problems. In this context, he described an almost perfect relationship with his girlfriend but, at the same time, behaved in a completely contrary manner. He endangered the relationship with his temporary promiscuous behavior, which he denied and repressed. From a psychodynamic perspective, the patient demonstrated infantile tendencies, including clear signs of identity diffusion, a disrupted concept of self and others, and a lack of care, concern, and responsibility for oneself. While *clarification* episodes during the interview prepared for identifying contradictions and conflicts, *confrontation* and *interpretation* episodes aimed to reveal splitting and denial mechanisms, uncovering that the patient’s repressed guilt feeling toward his father may hinder him from living a fulfilling life.

### 3.2. EEG results

**FIGURE 1:**
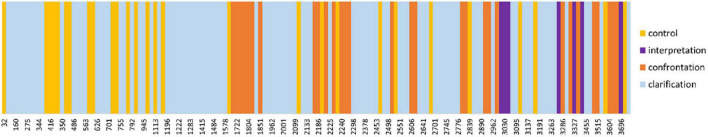
X-axis of the graphs shows the starting points with the color-coded conditions during the interview. Yellow, control; light blue, clarification; orange, confrontation; and violet, interpretation.

Based on the therapist’s interventions, we classified the patient’s artifact-free epochs of the EEG signal into “segments” according to intervention type: clarification, confrontation, interpretation, and a control condition. To obtain a precise estimate of the duration and distribution of the seconds/condition of the interview, we considered the total duration of the interview and for each condition in the first step and calculated the talking and listening to the therapist durations of the patient in the second step. The total duration of the clarification condition was 622 s and started at second 39. The confrontation condition lasted 293 s in total and started with a first interaction at minute 28 and 5 s. The interpretation segments started at minute 50 and 11 s with a total time of 102 s, and the control condition lasted 122 s, starting at second 33 at the beginning of the interview (see Figure 1).

In the clarification condition, the patient spoke for 560 s (47.37%) and listened to the interviewer for 622 s (52.62%). In the confrontation condition, the evaluation of speaking and listening durations showed that the patient spoke for 136 s (31.70%) and listened to the interviewer for 293 s (68.30%). In the interpretation condition, the patient spoke for 64 s (39.29%) and listened to the interviewer for 102 s (61.91%). In the control condition, both speaking and listening durations lasted 122 s.

Based on the sentences identified by the expert ratings, a total of 1139 s was determined for EEG analysis. Of these, 340 s were excluded due to artifacts, resulting in a maximum of 799 s available for the statistical analysis, which were, in turn, divided between the different conditions. This resulted in a final duration time for each of the conditions: The clarification condition comprised a total of 622 s. Of these, 434 s were again included in the statistical analysis, for which 188 s were excluded as artifacts. The confrontation condition involved 293 s, of which 218 s were included in the statistical analysis and 75 s were omitted due to artifacts. The interpretation condition comprised a total of 102 s, of which 71 s were used in the statistical analysis and 31 s were excluded as artifacts. The control condition comprised 122 s, with 46 s omitted due to artifacts and 76 s used for statistics. A quasibinomial logistic regression revealed no significant result between the intervention interpretation [β = −0.25, SE = 0.58, *t*(3,158) = −0.43, n.s], confrontation [β = −0.45, SE = 0.36, *t*(3,158) = −1.24, n.s], clarification [β = −0.28, SE = 0.29, *t*(3,158) = −0.99, n.s], and the control condition (see Table 1).

**TABLE 1:** Mean values, standard deviation (SD), min/max values, and subsegment counts (count seg.) of logarithms of alpha and beta power μV^2^ in the frontal (Fp1; Fp2), central (C3 and C4), and parieto-temporal (P3 and P4) electrodes (mean values averaged across subsegments for each condition).

	Mean (SD)	Min
**Alpha power**		
**Central electrodes**		
Control	1.78 (0.68)	−0.06
Clarification	1.64 (0.60)	−0.83
Confrontation	1.47 (0.62)	−0.70
Interpretation	1.33 (0.67)	−0.29
**Frontal electrodes**		
Control	2.37 (0.73)	0.41
Clarification	2.36 (0.76)	0.23
Confrontation	2.15 (0.70)	0.52
Interpretation	2.06 (0.77)	0.20
**Parieto-temporal electrodes**		
Control	2.32 (0.65)	0.56
Clarification	2.28 (0.67)	−0.18
Confrontation	2.18 (0.69)	−0.21
Interpretation	2.02 (0.65)	0.81
**Beta power**		
**Central electrodes**		
Control	0.59 (0.30)	−0.25
Clarification	0.51 (0.35)	−0.55
Confrontation	0.54 (0.34)	−0.41
Interpretation	0.61 (0.33)	−0.20
**Frontal electrodes**		
Control	1.37 (0.63)	−0.07
Clarification	1.32 (0.63)	−0.26
Confrontation	1.19 (0.61)	−0.60
Interpretation	1.17 (0.84)	−0.88
**Parieto-temporal electrodes**		
Control	0.90 (0.37)	−0.12
Clarification	0.70 (0.44)	−0.31
Confrontation	0.76 (0.46)	−0.57
Interpretation	0.68 (0.37)	−0.22

Participant’s electrophysiological responses in the EEG power spectrum were analyzed in the alpha and beta power in the frontal, central, and parietal electrodes in the different interventions (condition: Clarification, confrontation, interpretation, and control condition) and the brain lateralization (left/right hemisphere) (see Table 2).

**TABLE 2:** Report of the interview length in seconds for the interventions assessed by the experts.

	Total sec	Analyzed sec	Removed sec	% Included
All conditions	1139	799	340	70.15
Clarification	622	434	188	69.77
Confrontation	293	218	75	74.40
Interpretation	102	71	31	69.61
Control	122	76	46	62.30

The first column shows the seconds in total for the electroencephalographic (EEG) analysis (total sec), the second column shows the analyzed seconds after artifact rejection (analyzed sec), and the third column the rejected parts in seconds (removed sec). The last column shows the percentage of the included interview parts in seconds after artifact rejection (% included).

#### 3.2.1. Alpha power spectrum

**FIGURE 2:**
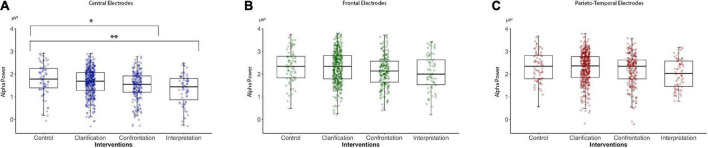
Logarithms of alpha band power (8–12.5 Hz, μV^2^) in the central, frontal, and parieto-temporal electrodes of psychoanalytic intervention techniques (clarification, confrontation, and interpretation) and a control condition recorded during a structural psychoanalytic interview: **(A)** Alpha power in central electrodes, **(B)** alpha power in frontal electrodes, and **(C)** alpha power in the parieto-temporal electrodes. *p < 0.05, **p < 0.01.

An omnibus test for the effect of intervention type in the *central electrodes* revealed an overall significant result in the alpha power spectrum [χ^2^(3) = 11.95, *p* = 0.007]. The intervention “interpretation” showed a significantly lower alpha power in comparison with the control condition [β = −0.41, SE = 0.15, *t*(147) = −2.80, *p* = 0.006] similar to the intervention “confrontation” [β = −0.28, SE = 0.11, *t*(147) = −2.59, *p* = 0.01]. By contrast, “clarification” showed no significant power difference to the control condition in the central electrodes [β = −0.11, SE = 0.09, *t*(147) = −1.14, n.s]. Results of the logarithms of alpha power in the central electrodes of the interventions clarification, confrontation, interventions and control condition can be seen in Figure 2A. Alpha power was higher in the left than in the right hemisphere in these electrodes [β = −0.37, SE = 0.04, *t*(1452) = −10.60, *p* < 0.001]. There was no interaction with the intervention type (see Figure 2).

To account for changes in alpha power that occurred for the interview, a model adjusted for time was calculated. After adjusting for time [β = −0.11, SE = 0.036, *t*(148) = −3.0, *p* = 0.0027], the “interpretation” intervention [β = −0.44, SE = 0.14, *t*(148) = −3.15, *p* = 0.002], and confrontation [β = −0.23, SE = 0.10, *t*(148) = −2.24, *p* = 0.026] scored significantly lower in alpha power compared to the control condition. The “Clarification” intervention [β = −0.06, SE = 0.10, *t*(148) = 0.52, n.s.] still showed no significant difference compared with the control condition in the central electrodes. Similar results were obtained without the adjustment.

The analysis of the alpha power spectrum in the *frontal electrodes* revealed a trend for an effect of intervention type in the omnibus test [χ^2^(3) = 9.58, *p* = 0.023]. While failing to reach stringent significance thresholds, the effects of intervention type relative to the control condition were similar to those of the central electrodes. Alpha power was lower in the condition “interpretation” than in the control condition [β = −0.25, SE = 0.14; *t*(147) = −1.71, *p* = 0.089] and during “confrontation” [β = −0.14, SE = 0.10; *t*(147) = −1.31, n.s.] but was no different than control during “clarification” [β = 0.05, SE = 0.09; *t*(147) = 0.53, n.s.]. Results of the logarithms of alpha power in the frontal electrodes of the interventions clarification, confrontation, interventions and control condition can be seen in Figure 2B. No hemispheric lateralization effect was found in the frontal electrodes [β = −0.04, SE = 0.04; *t*(1451) = −1.23, n.s.].

The analysis of the alpha power spectrum in the *parietal electrodes* failed to detect any significant effect of intervention type [χ^2^(3) = 3.43, n.s.] with no significant results in the intervention type interpretation [β = −0.23, SE = 0.16; *t*(147) = −1.41, n.s], confrontation [β = −0.03, SE = 0.11; *t*(147) = −0.24, n.s], and clarification [β = 0.03, SE = 0.1; *t*(147) = 0.75, n.s]. Results of the logarithms of alpha power in the parietal electrodes of the interventions clarification, confrontation, interventions and control condition can be seen in Figure 2C. However, there was a significant lateralization effect [β = −0.13, SE = 0.03; *t*(1452) = −3.90, *p* < 0.001]. There was no interaction with the intervention type.

#### 3.2.2. Beta power spectrum

Omnibus tests comparing a full model and a model without an intervention condition in the beta power gave no significant overall results in the central [χ^2^(3) = 2.27, n.s.], frontal [χ^2^(3) = 5.85, n.s], or parietal electrodes [χ^2^(3) = 5.50, n.s]. However, the planned comparisons relative to the control condition in the parietal electrodes exhibited an effect toward a lower beta spectral power in the condition “interpretation” [β = −0.22, SE = 0.10, *t*(147) = −2.11, *p* < 0.037], the interventions confrontation [β = −0.14, SE = 0.07, *t*(147) = −1.87, *p* < 0.064], and “clarification” [β = −0.11, SE = 0.06, *t*(147) = −1.75, *p* < 0.082]. Statistical significance failed after correction for multiple comparisons (see Figure 2).

## 4. Discussion

In this single-case study, we observed a decrease of power in the alpha band of a 32-electrode EEG during a psychoanalytic interview based on the technique of Kernberg (1981). This structured interview technique led to an emotional response, through which the lowered functional level of personality organization (borderline personality organization) with clear signs of identity diffusion, a disrupted concept of self, and a lack of care, concern, and responsibility for himself and others became evident in the interview. Interventional parts like confrontation and interpretation in a structured psychoanalytic interview and asking the patient to process personal unconscious conflicts have led to a destabilizing state and an impaired neurocognitive control mirrored by a loss in EEG alpha power.

In previous studies, decrements of alpha power in the EEG of awake participants were observed during the execution of cognitive tasks when subjected to the interference of external visual or acoustic signals or intervening limb movements. The visual signal is an externally evoked event that redirects EEG signals to posterior neuronal networks and can increase or decrease alpha power. For example, Pfurtscheller and Lopes da Silva (1999) described the basic principles of event-related EEG/MEG synchronization and desynchronization and that the other external motor sensor stimuli could have the same effect as visual stimuli. This result was confirmed by recent research by Hager et al. (2018). The decrements in alpha power of the present study suggest that the destructuring effect of confrontations and interpretations on this patient had an interfering effect on the patient’s autonomous mental processing. This conclusion is consistent with the finding that interpretations, which are potentially the most incisive interventions, had the strongest effects on alpha power. To our knowledge, this is the first attempt to examine if mutative psychoanalytic interventions, such as confrontations and especially interpretations, are associated with a modification of psychic processing in a patient as documented by the EEG.

Recent investigations with magnetic resonance imaging (MRI) and magnetoencephalogram (MEG) proved that the subcortical structures then interfere with cortical structures and show higher activity than during focused attention (Mazzetti et al., 2019). Neurophysiological studies showed that posterior neuronal oscillations in the alpha band (8–13 Hz) reflect the allocation of covert attention (Worden et al., 2000; Kelly et al., 2006; Thut et al., 2006; Jensen and Mazaheri, 2010) mediated by corticocortical interactions (Capotosto et al., 2012; Ptak, 2012; Vossel et al., 2014; Marshall et al., 2015a,b). Based on previous findings and theories that subcortical structures in the hypothalamus interfere with corticocortical neuronal structures during focused cognitive tasks and change the EEG rhythm (van Schouwenburg et al., 2010; Mazzetti et al., 2019), we may hypothesize that mutative interventions in a patient with an impaired personality structure are accompanied by a change in psychic processing with a correlate in the EEG power spectrum.

The strength of our approach was to measure for the first time a patient’s brain activity while being interviewed by an expert in the structural interview to identify expected differences in response to divergent psychoanalytic techniques. The main limitation of our study is that our findings are derived from one subject only. The results might be a very individual reaction of this one subject and dependent on age, gender, intellectual capacities of the subject, and other confounding variables like interaction with the interviewer and environmental factors. Moreover, we cannot completely rule out differences in motor behavior between the different conditions. It is possible that the patient was more agitated in one condition over the other and moved his leg(s) and/or hand(s) more relative to the control condition. This could possibly account for the reported differences observed over motor areas between the “interpretation” and “confrontation” and control conditions. Nevertheless, this single-case finding could serve as a first basis to repeat EEG recordings of a higher amount of psychoanalytic interviews with several subjects of different ages and gender. We may conclude that using EEG during therapeutic psychoanalytic techniques might be a helpful tool to evaluate differential responses to the process of psychodynamic psychotherapy.

## Data availability statement

The raw data supporting the conclusions of this article will be made available by the authors, without undue reservation.

## Ethics statement

The studies involving human participants were reviewed and approved by the Review Board of the University of Innsbruck. The patients/participants provided their written informed consent to participate in this study.

## Author contributions

AB and KL conceptualized the study together with PB, BS-U, and OK. KL conducted the EEG assessment. KL and FB analyzed the EEG data and performed statistical analyses. OK administered the structural interview with the patient and evaluated the structural diagnoses of the patient. AB and PB rated and segmented the episodes (clarification, confrontation, and interpretation) of the structural interview and described the interview process of the patient. BS-U and OK described the clinical diagnosis of the patient. NN gave valuable expert support for interpreting the EEG results. CP-S gave valuable support with respect to the EEG analyses. AB, KL, PB, and NN conceptualized the manuscript. AB and KL wrote the manuscript with contributions from all co-authors. All authors approved the final version of the manuscript and read and agreed to the published version of the manuscript.

## Footnotes

1
http://www.r-project.org/
